# Automated, image-based quantification of peroxisome characteristics with *perox-per-cell*

**DOI:** 10.1093/bioinformatics/btae442

**Published:** 2024-07-13

**Authors:** Maxwell L Neal, Nandini Shukla, Fred D Mast, Jean-Claude Farré, Therese M Pacio, Katelyn E Raney-Plourde, Sumedh Prasad, Suresh Subramani, John D Aitchison

**Affiliations:** Center for Global Infectious Disease Research, Seattle Children’s Research Institute, Seattle, WA 98109, United States; Department of Molecular Biology, School of Biological Sciences, University of California, San Diego, La Jolla, CA 92093, United States; Center for Global Infectious Disease Research, Seattle Children’s Research Institute, Seattle, WA 98109, United States; Department of Molecular Biology, School of Biological Sciences, University of California, San Diego, La Jolla, CA 92093, United States; Center for Global Infectious Disease Research, Seattle Children’s Research Institute, Seattle, WA 98109, United States; Department of Molecular Biology, School of Biological Sciences, University of California, San Diego, La Jolla, CA 92093, United States; Department of Molecular Biology, School of Biological Sciences, University of California, San Diego, La Jolla, CA 92093, United States; Department of Molecular Biology, School of Biological Sciences, University of California, San Diego, La Jolla, CA 92093, United States; Center for Global Infectious Disease Research, Seattle Children’s Research Institute, Seattle, WA 98109, United States; Departments of Pediatrics and Biochemistry, University of Washington, Seattle, WA 98195, United States

## Abstract

**Summary:**

*perox-per-cell* automates cumbersome, image-based data collection tasks often encountered in peroxisome research. The software processes microscopy images to quantify peroxisome features in yeast cells. It uses off-the-shelf image processing tools to automatically segment cells and peroxisomes and then outputs quantitative metrics including peroxisome counts per cell and spatial areas. In validation tests, we found that *perox-per-cell* output agrees well with manually quantified peroxisomal counts and cell instances, thereby enabling high-throughput quantification of peroxisomal characteristics.

**Availability and implementation:**

The software is coded in Python. Compiled executables and source code are available at https://github.com/AitchisonLab/perox-per-cell.

## 1 Introduction

Peroxisomes are membrane-bounded organelles found in all eukaryotic cells. Originally defined based on their production of hydrogen peroxide from oxidative reactions, peroxisomes perform various functions including fatty acid oxidation, lipid biosynthesis, and reactive oxygen species metabolism. Peroxisomes are produced in cells either through *de novo* biogenesis, where the organelle is assembled anew, or through growth and division. In humans, mutations in genes encoding proteins required for peroxisome biogenesis cause severe developmental defects, collectively referred to as the Zellweger spectrum of Peroxisome Biogenesis Disorders. While some of the molecular mechanisms underlying peroxisome biogenesis have been uncovered, our understanding of the process remains incomplete (Farré *et al.* 2019, Mast *et al.* 2020). Motivated by a need for high-throughput analyses to reveal critical components contributing to this process, we developed open-source software called *perox-per-cell* for image-based quantification of peroxisome characteristics in yeast cells. The software automates several time-consuming, traditionally manual image processing tasks including the segmentation of yeast cells and their peroxisomes as well as the collection of quantitative metrics such as the number of peroxisomes in individual cells, the spatial areas of cells and peroxisomes, and the luminal signal intensity within segmented peroxisomes. Through this automation, we have substantially increased the speed at which we can assess and quantify the impact of cellular and genetic perturbations on peroxisome number and size. Here, we detail the methods underlying *perox-per-cell* and demonstrate its validity for accurately quantifying metrics relevant for peroxisome research.

## 2 Materials and methods

*perox-per-cell* was developed to analyze microscopy data collected from yeast cells stained with Calcofluor white demarcating cell boundaries and producing green fluorescent protein (GFP) tagged with peroxisome targeting sequence 1 (PTS1) to locate peroxisomes. The software enables rapid assessment of peroxisomal phenotypes from 3D-imaging Z-stacks of the Calcofluor and GFP signals captured at the same physical position. With *perox-per-cell* we aimed to improve on previous work (Niemisto *et al.* 2006) and create a rigorously tested, publicly available tool for the research community.

**Figure 1.**
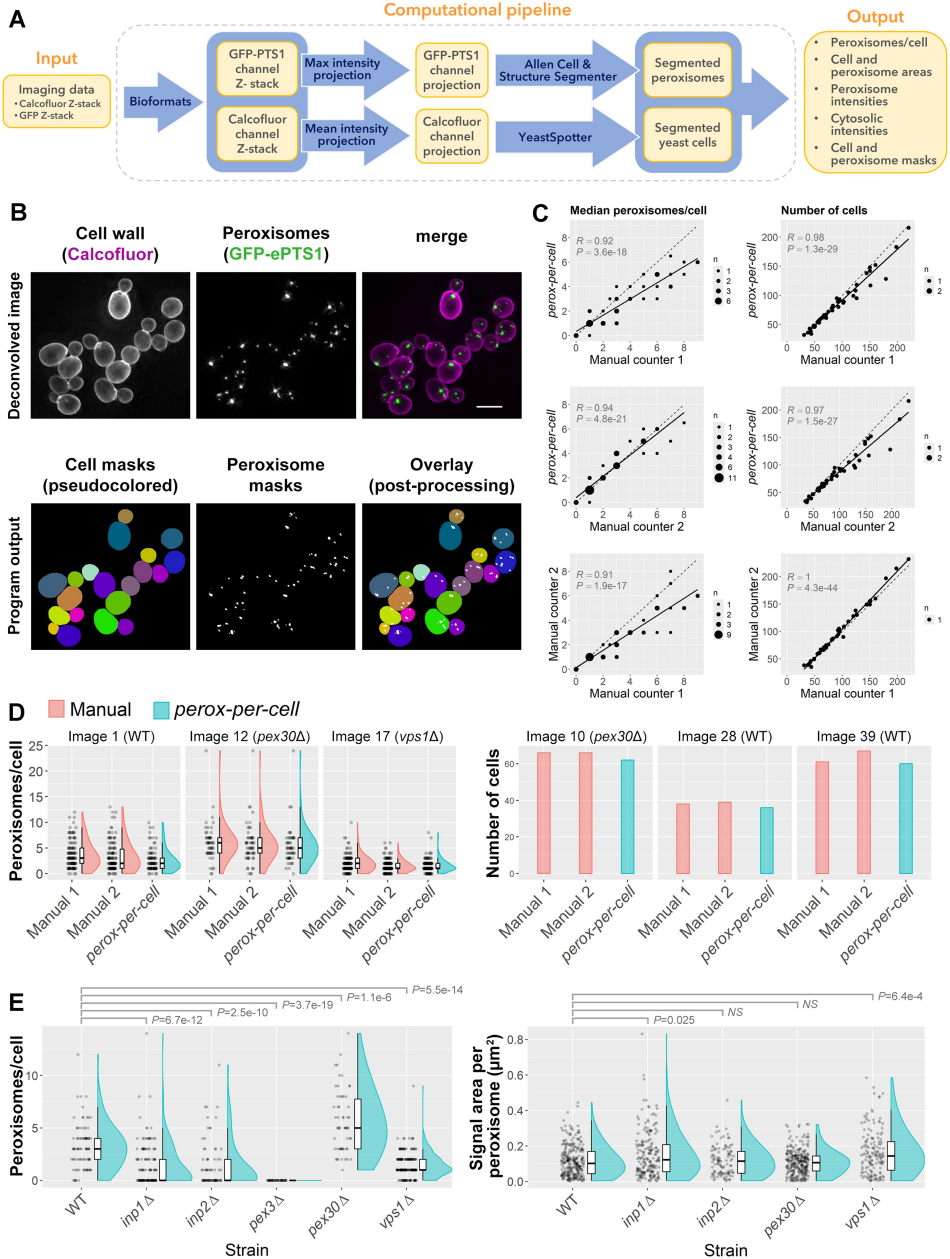
(A) Architecture of the *perox-per-cell* computational pipeline. (B) Example comparison of deconvolved images (top) used for manual counting and the cell and peroxisome masks generated by *perox-per-cell* (bottom). Scale bar: 5 μm. (C) Scatterplots with trendlines (solid) and unity lines (dashed) showing correlation between manual and auto-generated peroxisome counts (left) and number of cells (right) across test images. *R*, Pearson’s correlation coefficient; *P*, correlation *P*-value; *n*, number of observations at coordinates. (D) Distribution of peroxisome counts (left) and number of cells (right) derived manually or via *perox-per-cell* for three images where agreement between the methods was most representative of the overall agreement across test images. Distributions are shown using adjacent jitter, box, and half-violin plots. (E) Distribution of peroxisome counts (left) and areas (right) from images most representative of a given strain’s peroxisomal features across test images. FDR-adjusted *P*-values from Wilcoxon rank-sum tests comparing mutant strains to WT are shown above plots.

*perox-per-cell* first projects the Z-stacks onto a single 2D image plane for both the Calcofluor and GFP channels and then segments the cells and peroxisomes using automated tools. It then assigns peroxisomes to individual cells based on the overlap between the cell and peroxisome segmentation masks (Fig. 1A and B). Finally, the number of peroxisomes in each cell and additional metrics are output to an Excel spreadsheet.

### 2.1 Z-stack intensity projections

Imaging files are read into *perox-per-cell* using Bio-Formats (https://github.com/CellProfiler/python-bioformats). *perox-per-cell* uses file metadata to initialize 3D arrays of appropriate dimension to store the Z-stack intensity values in the Calcofluor and GFP channels. The program is designed to process input data consisting of two imaging channels, one for segmenting peroxisomes and the other for cells. After reading in the Z-stacks, a 2D maximum intensity projection is generated from the peroxisome channel Z-stack to ensure that each peroxisome, regardless of its position in the Z-dimension, is represented. The cell channel Z-stack is also converted into a 2D image so that it can be processed by the cell segmenter. For the cell channel, we use an *average* intensity projection because this resulted in more accurate segmentations in initial tests. Such projections may de-emphasize the presence of high intensity Calcofluor signals localizing on bud scars, generating a more even intensity profile along cell boundaries. We note that *perox-per-cell* can also process 2D images; a full 3D Z-stack is not required for either channel.

### 2.2 Cell segmentation

Several machine learning (ML) tools for automatic segmentation of yeast cells have become available in recent years (Lu *et al.* 2019, Dietler *et al.*, 2020, Salem *et al.* 2021). Based on visual inspection, applying the computational methods used by the YeastSpotter tool (Lu *et al.* 2019), which segments 2D images using a region-based convolutional neural network, gave sufficiently accurate segmentations. We initially chose YeastSpotter because it outperformed other established methods for segmenting fluorescently imaged cells (Lu *et al.* 2019). We also tested YeastNet (Salem *et al.* 2021); however, it was trained on brightfield images and did not identify any cells when applied to test images. We also compared YeastSpotter’s performance against the multiple prediction models available in Cellpose, a generalist, ML-based cell segmentation package (Stringer *et al.* 2021). When tested for accuracy against manual cell counts, results from YeastSpotter and the best performing Cellpose model (“cyto3”) gave comparable performance. To implement YeastSpotter segmentation, we adapted the code at https://github.com/alexxijielu/yeast_segmentation, only making a minor change to ensure our tool loads the underlying model weights correctly when deployed as a standalone application. After performing cell segmentation on the Calcofluor channel 2D projection, *perox-per-cell* saves the resulting image mask where all pixels corresponding to an individual cell share a unique intensity value.

### 2.3 Peroxisome segmentation

To segment peroxisomes, we adapted one of a suite of scripts in the Allen Cell & Structure Segmenter package (https://www.allencell.org/segmenter.html), which provides workflows for automated segmentation of various subcellular structures. After exploring example workflows in the imaging software Napari, we adapted the “gja1” workflow, which segments punctate features. Our workflow performs Gaussian smoothing on the 2D projection from the peroxisomal imaging Z-stack followed by spot detection via Laplacian of Gaussian filtering. Spot detection sensitivity is set via a user-defined input parameter that can be adjusted if too many, or too few, puncta are being detected as peroxisomes. By default, this parameter is set to a value that results in reasonable peroxisome segmentation based on initial test images of various yeast strains. An additional user-specified parameter sets the minimum peroxisome size, in pixels. Segmented peroxisomes below this size, which specifies the limit below which peroxisomes cannot be identified with high confidence, are removed from downstream analysis. Following these steps, a binary mask of the segmented peroxisomes is output, as well as a mask where pixels within individual peroxisomes share a unique label.

### 2.4 Quantification of peroxisome abundance and other metrics

To quantify the number of peroxisomes in each cell, *perox-per-cell* iterates over individual cells in the cell segmentation mask and counts the number of peroxisomes in the peroxisome segmentation mask found within each cell’s boundary. Cells with pixels on the image border are excluded because their full areas may not be represented, which can lead to undercounting. Each individual peroxisome is assigned to only one cell. If a peroxisome overlaps with multiple cells, it is assigned to the cell with the greatest overlap. In cases where a peroxisome’s maximum overlap is the same across multiple candidates, the peroxisome is assigned to the cell containing the greater signal intensity from the peroxisome channel projection image. If still unassigned after these steps, the peroxisome is randomly assigned to one of the candidates. The number of peroxisomes in each cell is tabulated and then output to a spreadsheet.

*perox-per-cell* also generates metrics for peroxisomal phenotypes, including the physical areas of each cell and peroxisome, the total summed intensity of the peroxisomal channel within cell boundaries, and the same summed intensity excluding signals from segmented peroxisomes. Accurate quantification of cell and peroxisomal physical areas requires that the physical size of pixels be specified in the input image metadata. To compute physical areas, *perox-per-cell* reads in metadata formatted according to the OME-XML standard (Goldberg *et al.* 2005) embedded in the input image. To accurately quantify areas, users should ensure that pixel width, height, and associated units are included in that metadata.

### 2.5 Software implementation, availability, and execution

The software is implemented in Python and available at https://github.com/AitchisonLab/perox-per-cell. We have also deployed *perox-per-cell* as a standalone, Windows executable package. When run, the software provides a GUI for selecting the imaging file(s) to process and for adjusting analysis parameters controlling peroxisome detection sensitivity, minimum peroxisome area, and the maximum possible intensity value in the peroxisomal channel. This last parameter should be set based on the bit depth of the peroxisome channel imaging data, equivalent to 2^(bit depth)^ – 1.

## 3 Results

To evaluate *perox-per-cell*, we compared its performance against manually quantified data. We collected 44 two-channel, 14-bit Z-stacks from various yeast strains including WT as well as *inp1*Δ, *inp2*Δ, *pex30*Δ, and *vps1*Δ mutants. These strains were selected because, together, they encompass a variety of peroxisomal densities and cellular distributions among strains relevant to peroxisome research. For example, *vps1*Δ cells predominantly have a single peroxisome whereas *pex30*Δ mutants show elevated peroxisome counts (Vizeacoumar *et al.* 2003, Hoepfner *et al.* 2001). Each imaging data set consisted of a Z-stack of Calcofluor-stained cells and a Z-stack from the same position to image GFP-PTS1 (see Supplementary methods). For manual counting, stacks were first deconvolved and then converted to maximum-intensity projections. Two individuals manually counted the number of peroxisomes in each cell from these images using blinded samples. We then ran *perox-per-cell* on the same imaging data and compared the results against manual counts. The imaging data used for this evaluation as well as the manual peroxisome counts are available at https://doi.org/10.5281/zenodo.11375101 (Neal *et al.* 2024).

### 3.1 Validation against manual peroxisome and cell counts

*perox-per-cell* results correlated strongly with manual counts (Fig. 1C, left and Supplementary Fig. S1). On average, the median number of peroxisomes per cell estimated by *perox-per-cell* in an image differed by -0.44 peroxisomes compared to the average of the two manual counters (Fig. 1D, left). Comparing *perox-per-cell* to manual counters 1 and 2 independently, this difference was -0.91 and 0.03, respectively. The difference between manual counters was 0.94, illustrating that *perox-per-cell* estimates fall within the variability that exists between manual counters. We also assessed the agreement between *perox-per-cell* and manual counts in terms of cell-to-cell variability. The average difference between the interquartile ranges (IQRs) of *perox-per-cell* and manual peroxisome counts was -0.33 across images. The IQR from manual counter 1 was 0.45 peroxisomes per cell higher than counter 2 on average, also indicating that software estimates fall within manual counter variability.

*perox-per-cell* also showed strong correlation with manual cell number counts (Fig. 1C, right and Supplementary Fig. S2). Across images, *perox-per-cell* detected a median value of 6.4% fewer cells (IQR = 6.5%) when compared to average counts recorded manually (Fig. 1D, right). Inspecting images with the largest discrepancies between manual and automated counts, we found that cells not detected by *perox-per-cell* tended to have lower signal intensities and were crowded by neighboring cells. We have also noticed that the cell segmentation tends to not detect very small buds, possibly due to relatively lower Calcofluor staining and, thus, lower signal intensity. We therefore encourage users to ensure that cell staining is sufficiently strong, and that cell crowding is minimal in input images.

### 3.2 Detecting altered peroxisome characteristics in mutants

We investigated whether the results from *perox-per-cell* could be used to detect statistically significant alterations in cellular peroxisome counts among mutant strains with peroxisomal defects. For each strain represented in our set of 44 test images, we selected a representative image for each strain (Supplementary methods), then compared peroxisome count distributions in mutant strains to WT. As part of this analysis, we also included a set of four images from a *pex3*Δ strain (Höhfeld *et al.* 1991) in which no peroxisomes were visually detected. We found that for all mutant strains tested, statistical comparisons against WT were significant with FDR-adjusted *P*-values ≤1.1e-06 for each strain (Fig. 1E, left).

We also tested whether peroxisome areas differed between strains and found that *inp1*Δ and *vps1*Δ had significantly higher areas compared to WT (Fig. 1E, right). We note that using the GFP channel intensities for this quantification is a proxy for peroxisomal area; differences in area could also represent differential PTS1 import. Together, these results indicate that *perox-per-cell* results can reveal statistically significant alterations in peroxisome characteristics in strains with known defects.

## 4 Discussion

Our results show that *perox-per-cell* accurately quantifies peroxisome counts from imaging data and that its estimates fall within the variability between manual counters. On a standard Windows workstation, the program processes images such as those used here in <90 s. Therefore, we anticipate it will be useful for rapidly and accurately assessing peroxisome characteristics in yeast cells, which, as we show here, can reveal statistically significant differences in peroxisome phenotypes between strains using one imaging instance per strain (Fig. 1E).

We note that *perox-per-cell* currently quantifies peroxisome characteristics from 2D projections derived from 3D Z-stacks, not directly from the Z-stacks themselves. Extending the software to support 3D segmentation could potentially improve the software’s accuracy and would add volumetric features to its list of quantifiable peroxisome characteristics. We also note that the peroxisome marker GFP-PTS1 used in this study is an exogenous reporter and that less abundant, endogenously-tagged proteins may result in dimmer puncta, possibly impacting peroxisome segmentation. We have found that in a strain endogenously producing GFP-tagged *PEX3*, *perox-per-cell* can produce accurate peroxisome segmentations despite dimmer puncta after adjusting the sensitivity parameter that controls peroxisome detection.

While Calcofluor staining was used to delineate cell boundaries in data used for this study, other staining methods may be applicable for use with *perox-per-cell*. The ML model used for cell segmentation was trained on fluorescent cell nuclei images; therefore, it may perform optimally for fluorescence imaging of more spherical cells. As additional studies are performed to assess the software’s generalizability across other cell types, staining methods, and image modalities, *perox-per-cell* can be enhanced with alternative methods and/or customization options for cell segmentation that broaden its applicability across the research community.

Additionally, the software’s peroxisome segmentation method is not specific for peroxisomes and could be used for segmenting other types of intracellular puncta provided that the user-defined parameters for peroxisome segmentation (see Section 2) are tuned accordingly.

With *perox-per-cell* we aim to facilitate peroxisome research in the domain of big data and its attendant analytical methods. The software opens new vistas for statistically powered investigations that establish or refine definitions of peroxisomal defects and that uncover novel phenotypes of known mutants unresolvable through coarse-grained analyses.

## Supplementary Material

btae442_Supplementary_Data

## Data Availability

The data used for tool validation are available in Zenodo at https://doi.org/10.5281/zenodo.11375101.

## References

[btae442-B1] Dietler N , Minder M, Gligorovski V et al A convolutional neural network segments yeast microscopy images with high accuracy. Nat Commun 2020;11:5723. 33184262 10.1038/s41467-020-19557-4 PMC7665014

[btae442-B2] Farré J-C , Mahalingam SS, Proietto M et al Peroxisome biogenesis, membrane contact sites, and quality control. EMBO Rep 2019;20:e46864. 30530632 10.15252/embr.201846864 PMC6322382

[btae442-B3] Goldberg IG , Allan C, Burel J-M et al The open microscopy environment (OME) data model and XML file: open tools for informatics and quantitative analysis in biological imaging. Genome Biol 2005;6:R47. 15892875 10.1186/gb-2005-6-5-r47 PMC1175959

[btae442-B4] Hoepfner D , van den Berg M, Philippsen P et al A role for Vps1p, actin, and the Myo2p motor in peroxisome abundance and inheritance in *Saccharomyces cerevisiae*. J Cell Biol 2001;155:979–90. 11733545 10.1083/jcb.200107028 PMC2150915

[btae442-B5] Höhfeld J , Veenhuis M, Kunau WH et al *PAS3*, a *Saccharomyces cerevisiae* gene encoding a peroxisomal integral membrane protein essential for peroxisome biogenesis. J Cell Biol 1991;114:1167–78. 1894692 10.1083/jcb.114.6.1167 PMC2289127

[btae442-B6] Lu AX , Zarin T, Hsu IS et al YeastSpotter: accurate and parameter-free web segmentation for microscopy images of yeast cells. Bioinformatics 2019;35:4525–7. 31095270 10.1093/bioinformatics/btz402 PMC6821424

[btae442-B7] Mast FD , Rachubinski RA, Aitchison JD. Peroxisome prognostications: exploring the birth, life, and death of an organelle. J. Cell Biol 2020;219:e201912100. 32211898 10.1083/jcb.201912100 PMC7054992

[btae442-B8] Neal M , Shukla N, Mast FD et al 2024. Manual quantification of peroxisome counts in yeast from 2-channel fluorescence Z-stacks [Data set]. *Zenodo*.

[btae442-B9] Niemisto A , Selinummi J, Saleem R et al. Extraction of the number of peroxisomes in yeast cells by automated image analysis. In: *2006 International Conference of the IEEE Engineering in Medicine and Biology Society*, 2353–2356, 2006. 10.1109/IEMBS.2006.259890 17945710

[btae442-B10] Salem D , Li Y, Xi P et al YeastNet: deep-learning-enabled accurate segmentation of budding yeast cells in Bright-Field microscopy. Appl. Sci 2021;11:2692.

[btae442-B11] Stringer C , Wang T, Michaelos M et al Cellpose: a generalist algorithm for cellular segmentation. Nat Methods 2021;18:100–6. 33318659 10.1038/s41592-020-01018-x

[btae442-B12] Vizeacoumar FJ , Torres-Guzman JC, Bouard D et al Pex30p, Pex31p, and Pex32p form a family of peroxisomal integral membrane proteins regulating peroxisome size and number in *Saccharomyces cerevisiae*. Mol Biol Cell 2003;15:665–77. 14617799 10.1091/mbc.E03-09-0681 PMC329287

